# Transcription factor EB (TFEB)-mediated autophagy protects bovine mammary epithelial cells against H_2_O_2_-induced oxidative damage *in vitro*

**DOI:** 10.1186/s40104-021-00561-7

**Published:** 2021-03-09

**Authors:** Xudong Sun, Renxu Chang, Yan Tang, Shengbin Luo, Chunhui Jiang, Hongdou Jia, Qiushi Xu, Zhihao Dong, Yusheng Liang, Juan J. Loor, Chuang Xu

**Affiliations:** 1 Heilongjiang Provincial Key Laboratory of Prevention and Control of Bovine Diseases, College of Animal Science and Veterinary Medicine, Heilongjiang Bayi Agricultural University, No. 5 Xinyang Road, Daqing, 163319 Heilongjiang Province China; 2 College of Veterinary Medicine, Hunan Agricultural University, Changsha, 410128 China; 3 Mammalian NutriPhysioGenomics, Department of Animal Sciences and Division of Nutritional Sciences, University of Illinois, Urbana, 61801 USA

**Keywords:** Apoptosis, Autophagy, Bovine mammary epithelial cells, Oxidative stress, TFEB

## Abstract

**Background:**

Bovine mammary epithelial cells after calving undergo serious metabolic challenges and oxidative stress both of which could compromise autophagy. Transcription factor EB (TFEB)-mediated autophagy is an important cytoprotective mechanism against oxidative stress. However, effects of TFEB-mediated autophagy on the oxidative stress of bovine mammary epithelial cells remain unknown. Therefore, the main aim of the study was to investigate the role of TFEB-mediated autophagy in bovine mammary epithelial cells experiencing oxidative stress.

**Results:**

H_2_O_2_ challenge of the bovine mammary epithelial cell MAC-T increased protein abundance of LC3-II, increased number of autophagosomes and autolysosomes while decreased protein abundance of p62. Inhibition of autophagy via bafilomycin A1 aggravated H_2_O_2_-induced reactive oxygen species (ROS) accumulation and apoptosis in MAC-T cells. Furthermore, H_2_O_2_ treatment triggered the translocation of TFEB into the nucleus. Knockdown of TFEB by siRNA reversed the effect of H_2_O_2_ on protein abundance of LC3-II and p62 as well as the number of autophagosomes and autolysosomes. Overexpression of TFEB activated autophagy and attenuated H_2_O_2_-induced ROS accumulation. Furthermore, TFEB overexpression attenuated H_2_O_2_-induced apoptosis by downregulating the caspase apoptotic pathway.

**Conclusions:**

Our results indicate that activation of TFEB mediated autophagy alleviates H_2_O_2_-induced oxidative damage by reducing ROS accumulation and inhibiting caspase-dependent apoptosis.

## Introduction

The transition from late-gestation to lactation is considered the most striking and challenging period in the lactation cycle of dairy cows. During this period, an increase in the energy and nutrient needs for lactation cannot be met by feed intake resulting in a period of negative energy balance [1, 2]. This triggers mobilization of body fat and a subsequent increase in blood concentrations of fatty acids which, at the cellular level, increase the production of reactive oxygen species (ROS) [3]. Excessive generation of ROS in a system could exceed the system’s capacity to neutralize and eliminate them leading to oxidative stress [4] that in turn contributes to production diseases in dairy cows [5, 6].

The bovine mammary epithelial cell undergoes serious metabolic challenges and excessive ROS production during the transition period. A large portion of this response is due to high metabolic priority of the mammary gland during early lactation [7]. Elevated intracellular levels of ROS induce mammary epithelial cell apoptosis, which contributes to the decrease in milk yield [8, 9]. This process is regulated by the cysteine protease family (caspase) apoptotic pathway. Caspase 9, the initiator caspase component of the apoptosome complex, can be activated by ROS, which in turn leads to activation of a caspase 3 initiating the caspase cascade leading to apoptosis [10]. Because apoptosis of bovine mammary epithelial cells is modulated by cellular redox state [11], developing potential strategies for maintaining redox balance is particularly important in terms of preventing oxidative damage.

Autophagy is an adaptive catabolic process by which cytoplasmic proteins and organelles are targeted to lysosomes for degradation [12]. Upon nutrient deprivation, autophagy is often up-regulated in response to metabolic stress and excessive organelle damage to facilitate aggregated protein removal and provide energy for cells [13]. Work with non-ruminants has verified that activation of autophagy is a crucial determinant of mechanisms that mitigate metabolic stress and its associated oxidative stress [14, 15]. Induction of autophagy is tightly regulated at the transcriptional level and involves the master regulator, transcription factor EB (TFEB). Activation of TFEB-mediated autophagy protect cellular against oxidative stress and metabolic stress [16, 17]. Interestingly, enhanced autophagy was observed in mammary gland of dairy cows with hyperketonemia during early lactation [18]. Thus, TFEB-mediated autophagy in bovine mammary epithelial cells might play a positive role in response to oxidative stress.

Because bovine mammary epithelial cells are likely subject to altered intracellular redox balance due to the high metabolic rates and TFEB-mediated autophagy is an important mechanism in the cellular defense against oxidative stress, we hypothesized that activation of TFEB-mediated autophagy protects bovine mammary epithelial cells against oxidative stress. Thus, the aim of the present study was to investigate the effects of TFEB-mediated autophagy on oxidative stress in bovine mammary epithelial cells by downregulating and upregulating TFEB via silencing using small interfering RNA (siRNA) or transfecting TFEB overexpression adenovirus under H_2_O_2_ conditions *in vitro*.

## Materials and methods

### Cell culture and treatment

The bovine mammary epithelial cells line MAC-T was cultured in Dulbecco’s modified eagle’s medium: nutrient mixture F-12 medium (DMEM/F-12, Grand Island, New York, USA) supplemented with 10 μg/L insulin (Sigma-Aldrich, St. Louis, MO, USA), 10% (v:v) fetal bovine serum (Grand Island, New York, USA), 100 U/mL penicillin (Hyclone, USA) and 100 g/mL streptomycin (Hyclone, USA) in a humidified incubator at 37 °C in an atmosphere of 5% CO_2_. The MAC-T cells were cultured with 0, 0.25, 0.5 or 1 mmol/L H_2_O_2_ for 24 h. Bafilomycin A1 (BafA1) was diluted in dimethylsulfoxide (DMSO) to a final working concentration of 100 μmol/L and DMSO was used as the vehicle control (Veh). MAC-T cells were pre-treated with BafA1 (100 nmol/L) or DMSO (vehicle control; Veh) in amounts equal to that present in BafA1 cultures for 12 h, and then treated with 0.5 mmol/L H_2_O_2_ for 24 h.

### RNA interference

The siRNA targeting TFEB and scrambled non-target negative control were designed and synthesized by Shanghai Genechem Co., Ltd. (Shanghai, China). The TFEB-siRNA primer sequence was sense: 5'-GCAUUACAUGCAGCAGCAAdTdT-3' and antisense: 5'-UUGCUGCUGCAUGUAAUGCdTdT-3'. The scrambled negative control siRNA primer sequence was sense: 5'-UUCUCCGAACGUGUCACGUdTd-3' and antisense: 5'-ACGUGACACGUUCGGAGAAdTd-3'. MAC-T cells were seeded into six-well plates (2 × 10^6^ cells per well) and cultured in serum-free, antibiotic-free DMEM/F12 medium overnight. The siRNA was diluted in serum-free DMEM/F12 medium and then mixed with Lipofectamine 2000 (Invitrogen, Carlsbad, CA). After incubation for 10 min, the siRNA/Lipofectamine solution was added directly to cells with serum-free, antibiotic-free DMEM/F12 medium for 6 h. The final siRNA concentration was 50 nmol/L. Medium was switched to DMEM/F-12 medium supplemented with 10 μg/L insulin, 10% (v:v) fetal bovine serum, 100 U/mL penicillin and 100 g/mL streptomycin for 42 h. Cells were used for subsequent analysis or treatment.

### Adenovirus transfections

Empty adenoviral vector (EV, 3.16 × 10^10^ plaque-forming units/mL) and TFEB overexpression adenovirus (Ad-TFEB, 3.16 × 10^10^ plaque-forming units/mL) were constructed by Hanbio (Shanghai, China). In brief, full-length TFEB was amplified using PCR and subcloned into the pAdEasy-EF1-MCS-CMV vector. The PCR primer sequence was sense: 5'-TGTGACCGGCGCCTACTCTGGTACCGCCACCATGGCGTCTCGAAT-3' and antisense: 5'-TCTTATCTAGAAGCTTAGGCTCGAGTCACAGCACGTCGCCCTCCT-3'. Recombinant adenovirus mRFP-GFP-LC3 (1 × 10^10^ plaque-forming units/mL) was purchased from Hanbio (HB-AP2100001, Shanghai, China). In brief, full-length LC3 was amplified using PCR and conjugated to mRFP-GFP. It was then subcloned into the pHBAd-CMV vector. The PCR primer sequence was sense: 5'-CGCAAATGGGCGGTAGGCGTG-3' and antisense: 5'-AAACCACAACTAGAATGCAGT-3'. Adenovirus was transfected into MAC-T cells according to the manufacturer’s instructions. MAC-T cells were transfected with 50 multiplicity of infection of adenovirus in serum-free, antibiotic-free DMEM/F12 medium for 6 h. Medium was then switched to DMEM/F-12 medium supplemented with 10 μg/L insulin, 10% (v:v) fetal bovine serum, 100 U/mL penicillin and 100 g/mL streptomycin for 42 h. After transfection, cells were treated with or without 0.5 mmol/L H_2_O_2_ for 24 h. After washing with phosphate-buffered saline (PBS) and fixation in 4% paraformaldehyde, nuclei were stained with 4′,6-diamidino-2-phenylindole dihydrochloride (DAPI) (D8417; Sigma-Aldrich). Subsequently, cells were imaged using an Olympus FLUOVIEW FV1000 microscope.

### Immunofluorescence staining

Immunofluorescence analysis was done according to a published procedure [19]. After treating as indicated, MAC-T cells were fixed in 4% paraformaldehyde at room temperature for 30 min, followed by washing 3 times with PBS. Cells were then incubated 0.1% Triton X-100 (T9284; Sigma-Aldrich, St. Louis, MO, USA) for 10 min at room temperature and washed 3 times with PBS. After antigen retrieval using EDTA-Na_2_ (1 mmol/L) at 95 °C for 5 min, cells were incubated at 4 °C overnight with rabbit primary antibody for TFEB (13372-1-AP, Proteintech, Rosemont, IL, USA; 1:50) at 4 °C overnight. After washing 3 times with PBS, cells were incubated with goat anti-rabbit IgG conjugated with cy3 (A0516, Beyotime Institute of Biotechnology, Jiangsu, China, 1:200). After washing 3 times with PBS, cells stained with DAPI (10 μg/mL) (D8417, Sigma-Aldrich) at room temperature for 10 min. After washing 3 times with PBS, cells were imaged using an Olympus FLUOVIEW FV1000 microscope.

### Determination of intracellular ROS

The peroxide sensitive fluorescent probe 2′7’-dichlorofluorescein diacetate (Beyotime Institute of Biotechnology) was used to detect intracellular ROS according to a previous study [20]. After treatment as indicated, MAC-T cells were incubated with 25 μmol/L 2′,7'-dichlorofluorescein diacetate in serum-free DMEM/F12 medium at 37 °C for 20 min. Cells were then washed twice with PBS and resuspended with serum-free DMEM/F12 medium. The fluorescence was analyzed by flow cytometry (FACSCalibur, Becton-Dickinson, Sunnyvale, CA, USA). Results were expressed as fold changes by normalizing the data to the control values.

### Measurement of cells apoptosis

Cell apoptosis was detected using a propidium iodide and annexin V-FITC apoptosis detection kit (BD PharMingen, San Jose, CA, USA) following the supplier’s protocols. Briefly, after treatment as indicated, MAC-T cells were harvested with trypsin and washed twice with PBS. Cells were resuspended with 300 μL 1× Binding Buffer and stained with 10 μL Annexin V-FITC for 15 min in dark at room temperature. Then, cells were stained with 5 μL PI for 5 min. After staining, cells were mixed with 200 μL 1× Binding Buffer and analyzed by flow cytometry (Becton Dickinson, San Jose, CA, USA). Number of apoptotic cells were calculated as percentages of total cells.

### Total RNA extraction and quantitative reverse-transcription PCR

Total RNA from the MAC-T cells was isolated with RNAiso Plus (TaKaRa Biotechnology Co. Ltd., Dalian, China) according to protocols from the manufacturer. Concentration of RNA was determined with a K5500 MicroSpectrophotometer (Beijing Kaiao Technology Development Ltd., Beijing, China), and the integrity number of the RNA was determined using the Agilent 2100 bioanalyzer (Agilent Technologies, Santa Clara, CA). All samples had an RNA integrity number factor greater than 8.0. For cDNA, 1 μg of total RNA was reverse transcribed using a reverse transcription kit (RR047A, TaKaRa Biotechnology Co., Ltd.) following instructions from the manufacturer. Quantitative reverse-transcription PCR was performed using SYBR green plus reagent kit (Roche, Norwalk, CT, USA) with the 7500 Real-Time PCR System (Applied Biosystems) as described previously [20]. The final primer concentration was 10 μmol/L. Primer sequences are reported in Table 1. Gene abundance was calculated by the 2^–ΔΔCT^ method using the GAPDH and Ubiquitin B as internal controls.

**Table 1:** Primer sequences of the genes analyzed

Gene	Primer sequences (5′→3′)	Length, bp	Efficiency (E%)
*TFEB*	F TGCTGACCCCAGATCCAACT R CCCAAACCTGCTTGATCACC	76	101
β-actin	F GCCCTGAGGCTCTCTTCCA R GCGGATGTCGACGTCACA	101	97
*GAPDH*	F GTCTTCACTACCATGGAGAAGG R TCATGGATGACCTTGGCCAG	197	103

The efficiency was determined as [E = (10^–1/slope^−1) × 100%]

### Protein extraction and western blotting

Total and nuclear proteins were extracted from cells with a nuclear and cytoplasmic protein extraction kit (P0013, P0027; Beyotime Institute of Biotechnology) according to protocols from the supplier. Concentration of protein was quantified with the bicinchoninic acid protein assay kit (P1511; Applygen Technologies Inc.). Sodium dodecyl sulfate polyacrylamide gel electrophoresis was performed to separate 30 μg sample protein, which were then transferred onto polyvinylidene difluoride (PVDF) membranes and blocked with Tris-buffered saline (TBST; 50 mmol/L Tris, pH 7.6, 150 mmol/L NaCl, and 0.1% Tween 20) supplemented with 3% BSA at room temperature for 4 h. The PVDF membranes were then incubated with antibodies against p62 (ab101266, Abcam, Cambridge, MA; 1:1000), LC3 (ab48394, Abcam, 1:1000), TFEB (13372–1-AP, Proteintech, Rosemont, IL, USA; 1:1000), Bax (ab32503, Abcam, 1:1000), Bcl-2 (ab201566, Abcam, 1:500), caspase 3 (ab4051, Abcam, 1:500), caspase 9 (sc-56,076, Santa Cruz, CA, USA, 1:1000), histone H3 (4499, Cell Signaling Technology Danvers, MA; 1:1000), and β-actin (ab8226, Abcam, 1:2000) overnight at 4°C. After incubating with primary antibody, membranes were washed and incubated with horseradish peroxidase-conjugated anti-mouse or anti-rabbit antibody (Boster, Wuhan, China) for 45 min at room temperature. Immunoreactive bands were visualized with an enhanced chemiluminescence solution (ECL, Millipore, Bedford, MA, USA) and scanned using a simple protein imager (ProteinSimple, Santa Clara, CA, USA). Band intensities were quantified by Image-Pro Plus 6.0 software (Media Cybernetics Inc., Rockville, MD, USA).

### Statistical analysis

Three replicate cultures were run for each treatment in each experiment, and each of the determinations was performed 3 times for each treatment. All data analyses were performed using GraphPad Prism 7 (GraphPad InStat Software, San Diego, CA, USA) and assessed for normality of distribution using the Shapiro-Wilk test. A Student’s *t*-test was performed for 2 group comparisons and one-way ANOVA followed by a Bonferroni correction was used to determine statistical significance for multiple comparisons. All data are reported as means ± standard error of the mean (mean ± SEM). Significance was set at *P* < 0.05.

## Results

### H_2_O_2_-induced oxidative stress and enhanced autophagy in MAC-T cells

**Fig. 1:**
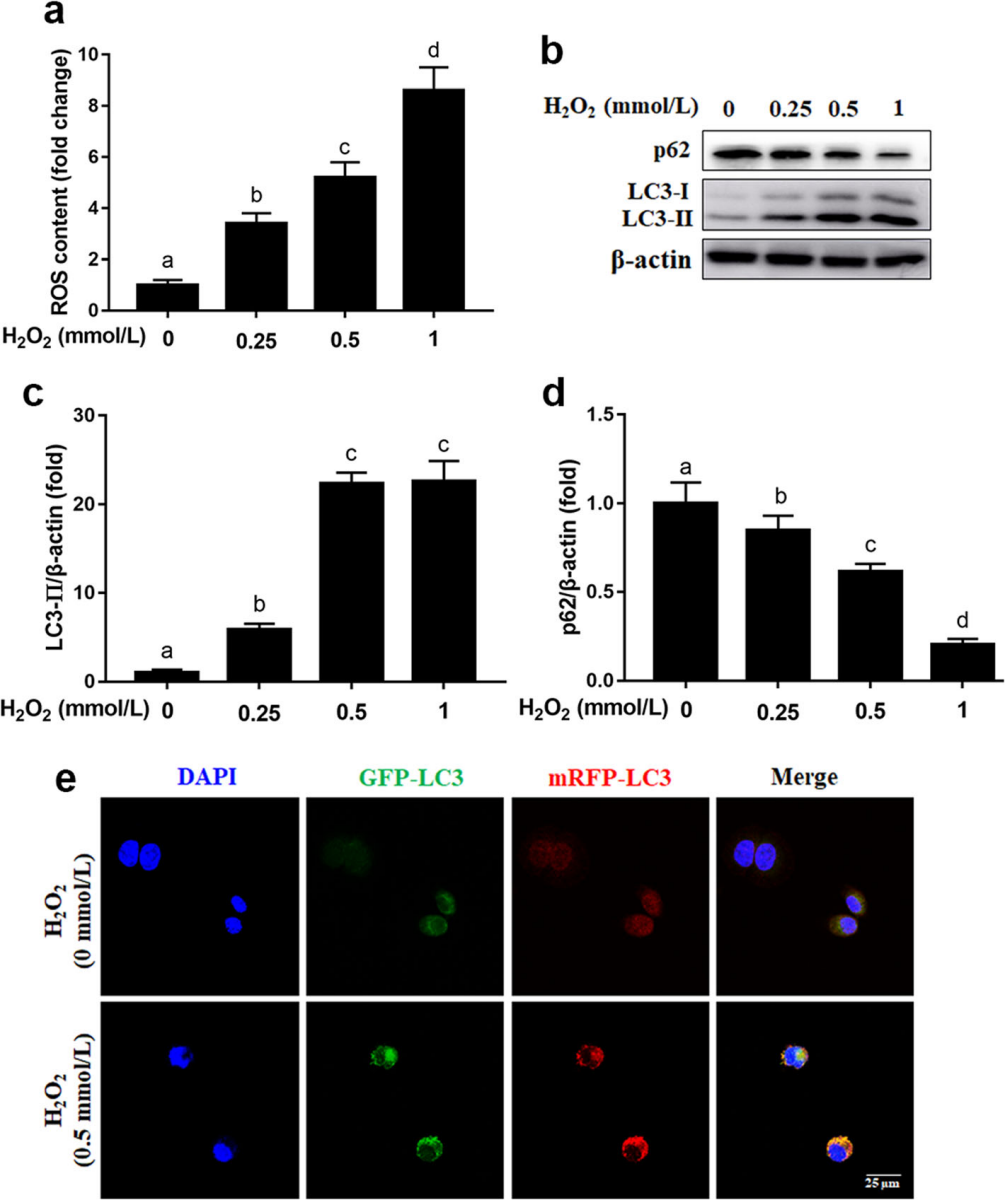
Effects of H_2_O_2_ on the oxidative stress and autophagy in MAC-T cells. **a** ROS content. MAC-T cells were treated with 0, 0.25, 0.5 or 1 mmol/L H_2_O_2_ for 24 h. **b** Western blot analysis of LC3-II and p62. **c** Protein abundance of LC3-II. **d** Protein abundance of p62. **e** Representative images of autophagosomes (yellow puncta) and autolysosomes (red puncta), scale bar = 25 μm. MAC-T cells were transfected with the recombinant adenovirus mRFP-GFP-LC3 for 48 h, and/or treated with H_2_O_2_ (0.5 mmol/L) for another 24 h. The data presented are the mean ± SEM. Different superscript lowercase letters in bar charts represent significant difference (*P* < 0.05)

Compared with the 0 mmol/L H_2_O_2_ group, 0.25, 0.5 or 1 mmol/L H_2_O_2_ enhanced the level of intracellular ROS in MAC-T cells (*P* < 0.05, Fig. 1a). Compared with the 0 mmol/L H_2_O_2_ group, protein abundance of LC3-II was greater in the 0.25, 0.5 or 1 mmol/L H_2_O_2_ group (*P* < 0.05, Fig. 1b and c). However, compared with the 0 mmol/L H_2_O_2_ group, protein abundance of p62 was lower in the 0.25, 0.5 or 1 mmol/L H_2_O_2_ group (*P* < 0.05, Fig. 1b and d). To measure formation of autophagosomes (yellow puncta) and autolysosomes (red puncta), recombinant adenovirus of mRFP-GFP-LC3 was used in H_2_O_2_-treated cells. Compared with 0 mmol/L H_2_O_2_, the 0.5 mmol/L H_2_O_2_ markedly increased the number of autophagosomes labeled with yellow puncta and autolysosomes labeled with red puncta (Fig. 1e).

### Inhibition of autophagy aggravated H_2_O_2_-induced oxidative damage in MAC-T cells

**Fig. 2:**
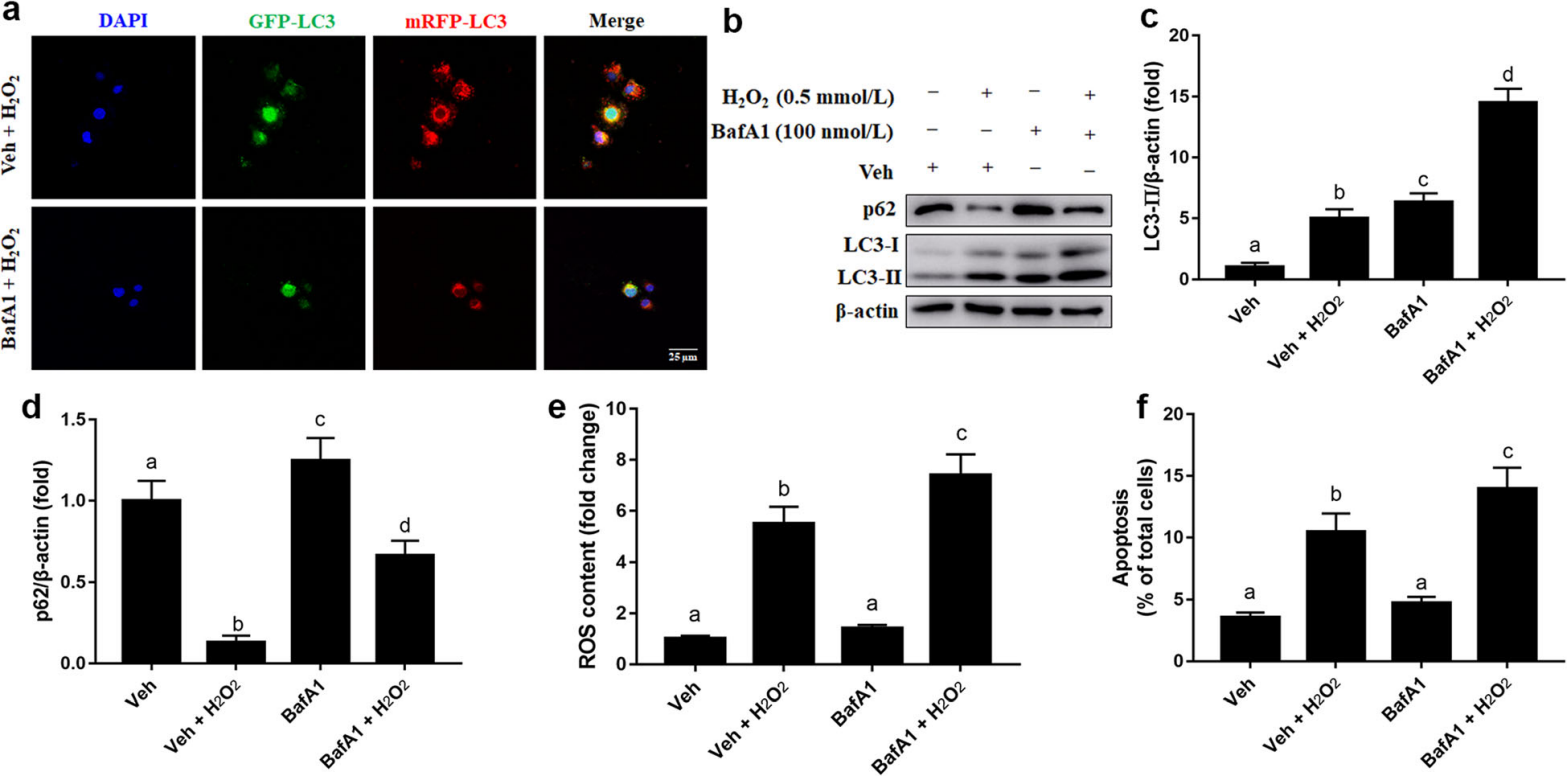
Effects of inhibition of autophagy on oxidative damage of MAC-T cells. **a** Representative images of autophagosomes (yellow puncta) and autolysosomes (red puncta), scale bar = 25 μm. MAC-T cells were transfected with recombinant adenovirus mRFP-GFP-LC3 for 48 h and then pre-treated with BafA1 (100 nmol/L) or DMSO (vehicle control; Veh) in amounts equal to that present in BafA1 cultures for 12 h, and treated with H_2_O_2_ (0.5 mmol/L) for another 24 h. **b** Western blot analysis of LC3-II and p62. MAC-T cells were pre-treated with BafA1 (100 nmol/L) or DMSO (Veh) in amounts equal to that present in BafA1 cultures for 12 h, and/or treated with H_2_O_2_ (0.5 mmol/L) for another 24 h. **c** Protein abundance of LC3-II. **d** Protein abundance of p62. **e** ROS content. **f** The % of apoptotic cells. The data presented are the mean ± SEM. Different superscript lowercase letters in bar charts represent significant difference (*P* < 0.05)

To evaluate effects of autophagy on oxidative stress, cells were pretreated with autophagy inhibitor BafA1 for 12 h and then treated H_2_O_2_ for 24 h. Compared with the Veh + H_2_O_2_ group, the number of autolysosomes labeled with red puncta was lower in the BafA1 + H_2_O_2_ group (Fig. 2a). Compared with the Veh group, protein abundance of LC3-II and p62 was greater in the BafA1 group (*P* < 0.05, Fig. 2b-d). Compared with the Veh + H_2_O_2_ group, protein abundance of LC3-II and p62 was greater in the BafA1 + H_2_O_2_ group (*P* < 0.05, Fig. 2b-d). Compared with the Veh + H_2_O_2_ group, ROS content was greater in the BafA1 + H_2_O_2_ group (*P* < 0.05, Fig. 2e). Furthermore, compared with the Veh + H_2_O_2_ group, cellular apoptosis was greater in the BafA1 + H_2_O_2_ group (*P* < 0.05, Fig. 2f).

### H_2_O_2_ triggered translocation of TFEB into the nucleus in MAC-T cells

**Fig. 3:**
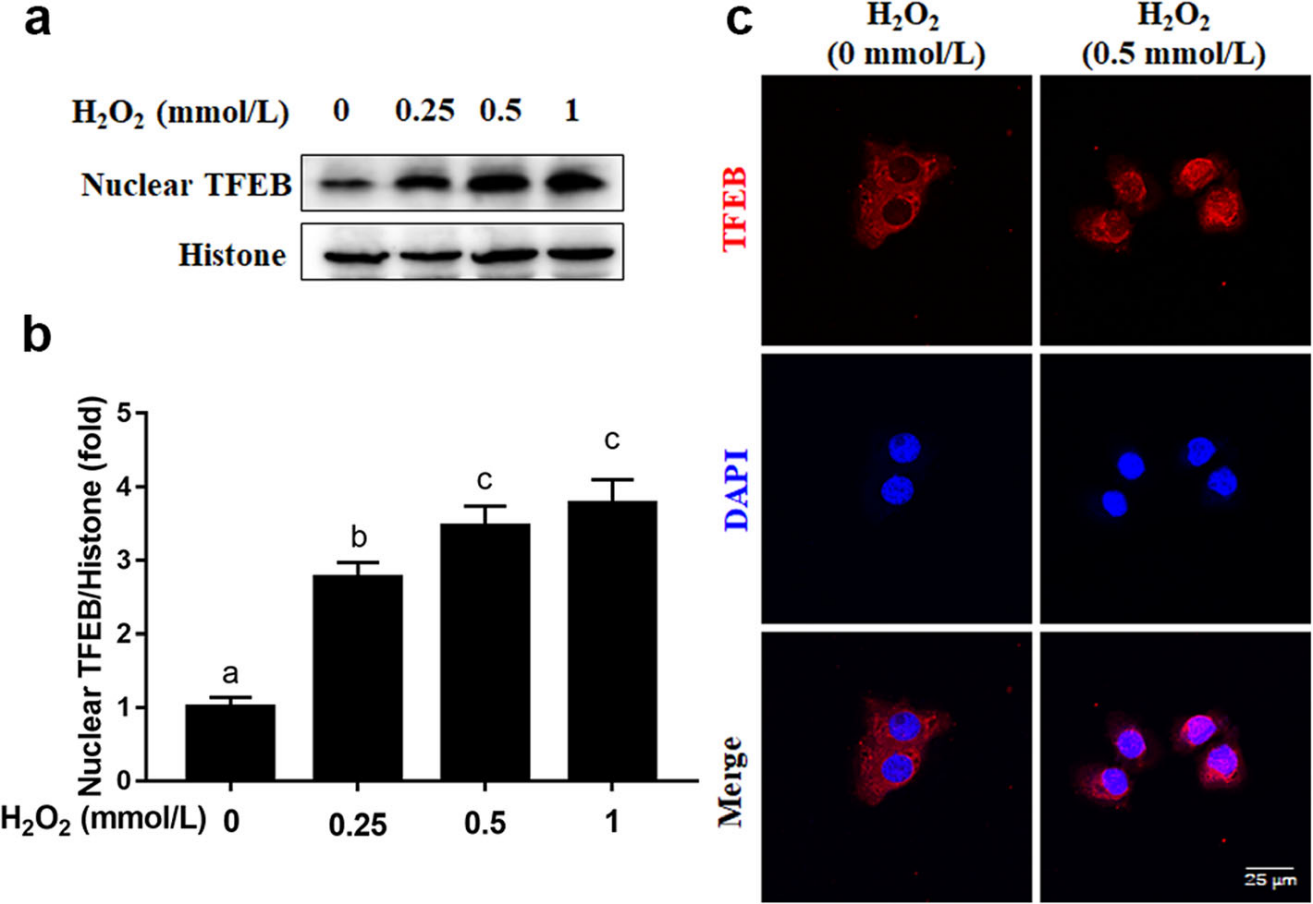
Effects of H_2_O_2_ on the location of TFEB in MAC-T cells. MAC-T cells were treated with 0, 0.25, 0.5 or 1 mmol/L H_2_O_2_ for 24 h. **a** Western blots analysis of nuclear TFEB. **b** Protein abundance of nuclear TFEB. **c** Immunofluorescence for TFEB (red) was performed, and the nuclear dye DAPI (blue) was used, scale bar = 25 μm. The data presented are the mean ± SEM. Different superscript lowercase letters in bar charts represent significant difference (*P* < 0.05)

Compared with the 0 mmol/L H_2_O_2_ group, nuclear protein abundance of TFEB was greater in the 0.25, 0.5 or 1 mmol/L H_2_O_2_ group (*P* < 0.05, Fig. 3a and b). Consistent with the alterations in nuclear protein abundance of TFEB, immunofluorescence staining results revealed that H_2_O_2_ treatment induced TFEB translocation to the nucleus (Fig. 3c).

### Knockdown of TFEB reversed H_2_O_2_-induced autophagy in MAC-T cells

**Fig. 4:**
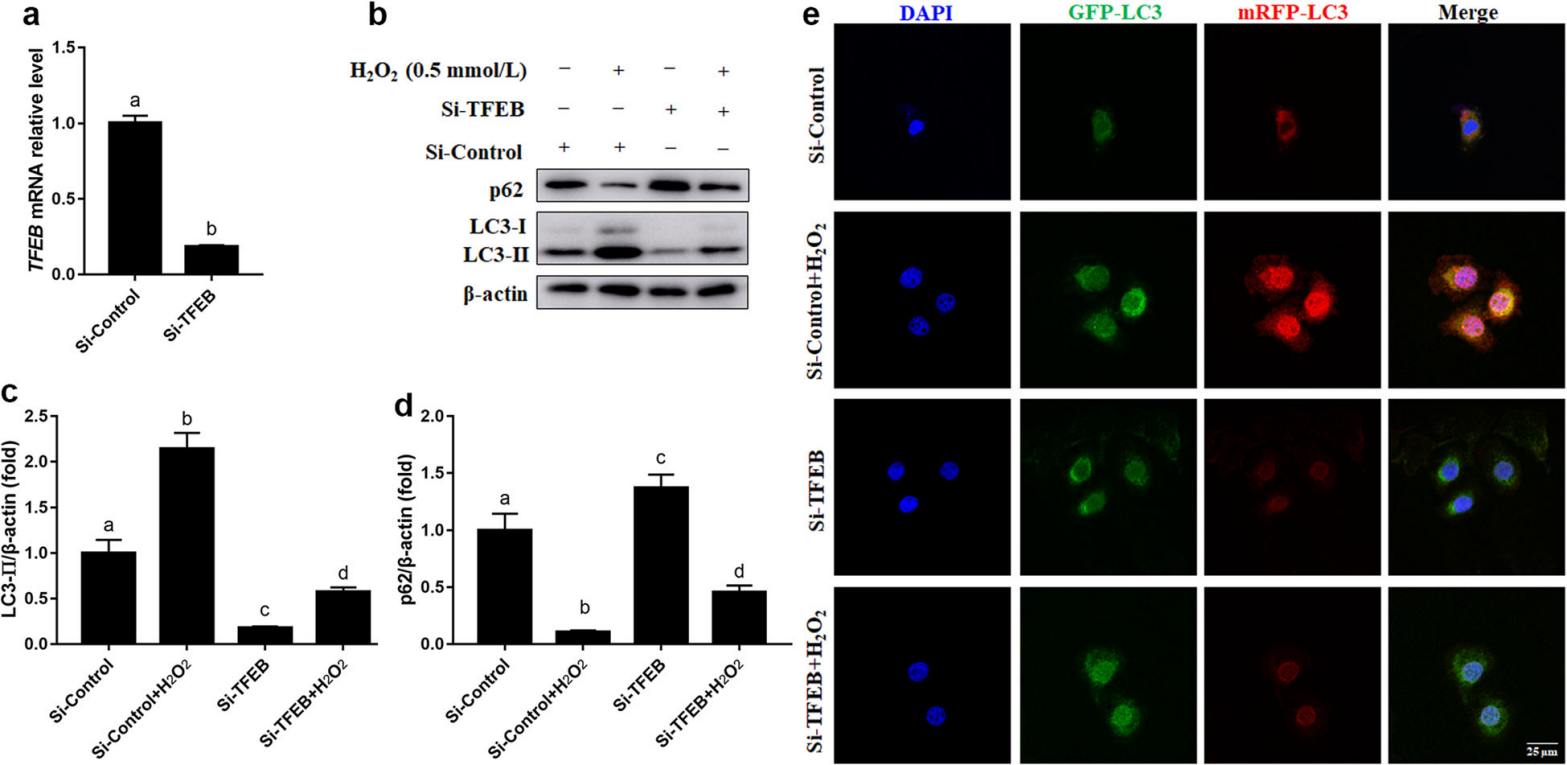
Effects of TFEB knockdown on autophagy in MAC-T cells. **a** mRNA abundance of TFEB. MAC-T cells were transfected with TFEB siRNA for 48 h. **b** Western blot analysis of LC3-II and p62. MAC-T cells were transfected with TFEB siRNA for 48 h and/or treated with H_2_O_2_ (0.5 mmol/L) for another 24 h. **c** Protein abundance of LC3-II. **d** Protein abundance of p62. **e** Representative images of autophagosomes (yellow puncta) and autolysosomes (red puncta), scale bar = 25 μm. MAC-T cells were co-transfected with the recombinant adenovirus mRFP-GFP-LC3 and TFEB siRNA for 48 h, and/or treated with H_2_O_2_ (0.5 mmol/L) for another 24 h. The data presented are the mean ± SEM. Different superscript lowercase letters in bar charts represent significant difference (*P* < 0.05)

Compared with the siControl group, mRNA abundance of TFEB was lower in the siTFEB group (*P* < 0.05, Fig. 4a). Knockdown of TFEB decreased the protein abundance of LC3-II and attenuated the upregulation of protein abundance of LC3-II induced by H_2_O_2_ (*P* < 0.05, Fig. 4b and c). In addition, knockdown of TFEB increased protein abundance of p62 and attenuated the downregulation of protein abundance of p62 induced by H_2_O_2_ (*P* < 0.05, Fig. 4b and d). Consistent with alterations in protein abundance of LC3-II, knockdown of TFEB decreased the number of autophagosomes labeled with yellow puncta and autolysosomes labeled with red puncta and attenuated the increase in number of autophagosomes and autolysosomes induced by H_2_O_2_ (Fig. 4e).

### Overexpression of TFEB attenuated H_2_O_2_-induced ROS accumulation in MAC-T cells

**Fig. 5:**
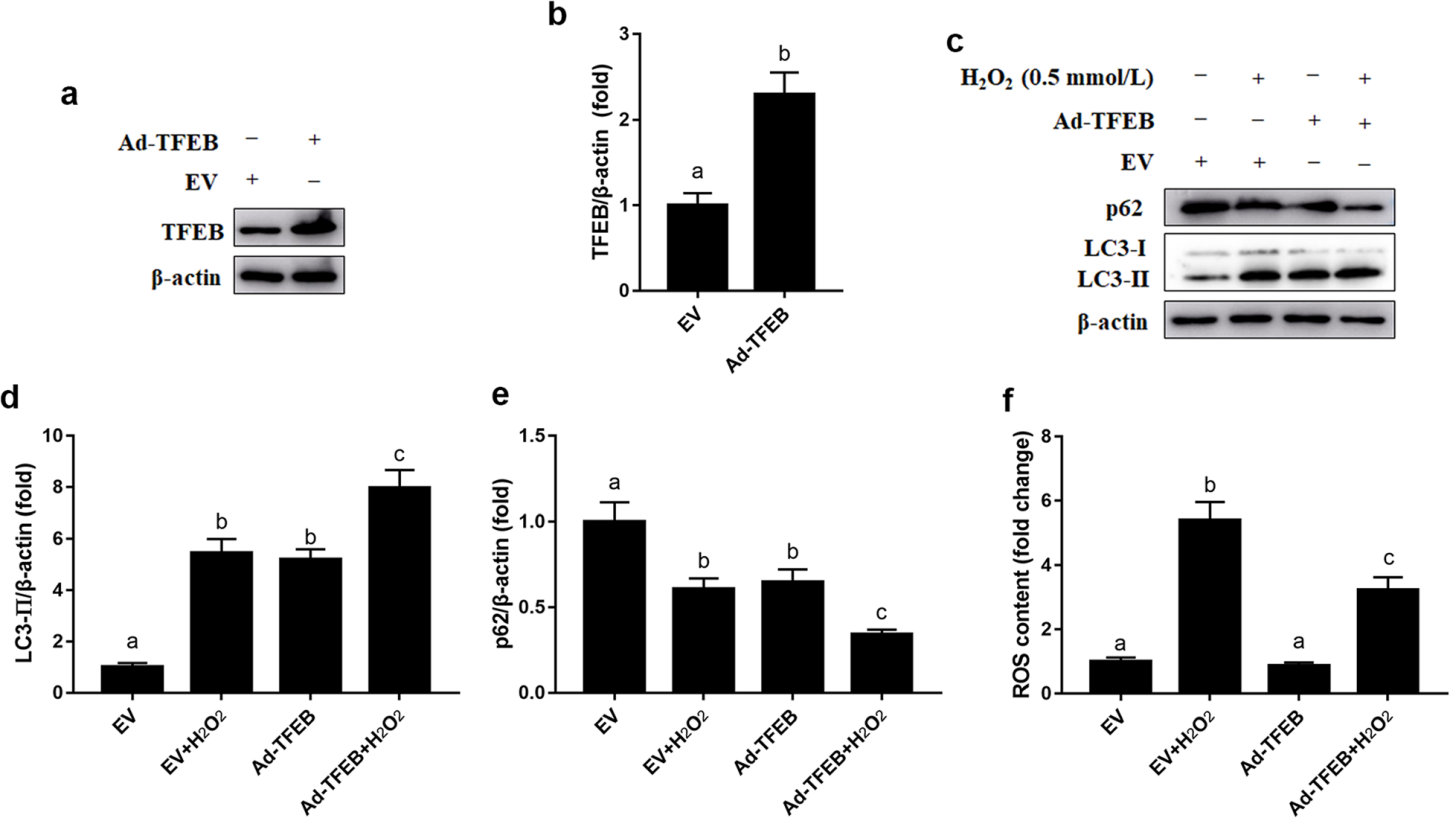
Effects of TFEB overexpression on ROS accumulation in MAC-T cells. **a** Western blots analysis of TFEB. MAC-T cells were transfected with TFEB overexpression adenovirus for 48 h. **b** Protein abundance of TFEB. **c** Western blots analysis of LC3-II and p62. MAC-T cells were transfected with TFEB overexpression adenovirus for 48 h and/or treated with H_2_O_2_ (0.5 mmol/L) for another 24 h. **d** Protein abundance of LC3-II. **e** Protein abundance of p62. **f** ROS content. The data presented are the mean ± SEM. Different superscript lowercase letters in bar charts represent significant difference (*P* < 0.05)

Compared with the EV group, protein abundance of TFEB was greater in the Ad-TFEB group (*P* < 0.05, Fig. 5a and b). Overexpression of TFEB increased protein abundance of LC3-II (*P* < 0.05, Fig. 5c and d), while it decreased protein abundance of p62 (*P* < 0.05, Fig. 5c and e). Furthermore, TFEB overexpression decreased overall ROS content and attenuated the increase in ROS content induced by H_2_O_2_ (*P* < 0.05, Fig. 5f).

### Overexpression of TFEB attenuated H_2_O_2_-induced apoptosis in MAC-T cells

**Fig. 6:**
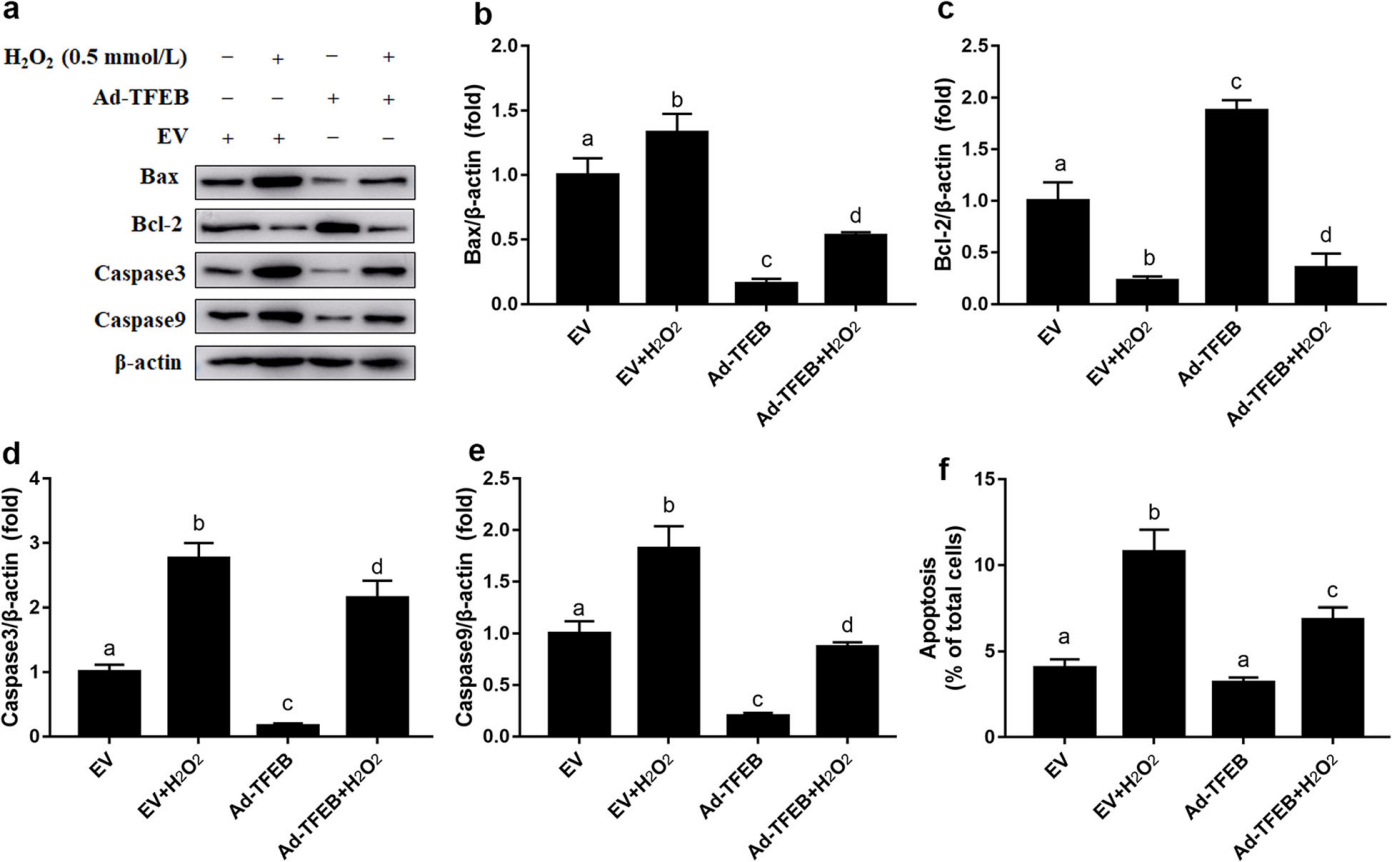
Effects of TFEB overexpression on apoptosis of MAC-T cells. MAC-T cells were transfected with TFEB overexpression adenovirus for 48 h and/or treated with H_2_O_2_ (0.5 mmol/L) for another 24 h. **a** Western blots analysis of Bax, Bcl-2, caspase 3 and caspase 9. **b** Protein abundance of Bax. **c** Protein abundance of Bcl-2. **d** Protein abundance of caspase 3. **e** Protein abundance of caspase 9. **f** The % of apoptotic cells. The data presented are the mean ± SEM. Different superscript lowercase letters in bar charts represent significant difference (*P* < 0.05)

Compared with the EV group, protein abundance of Bcl-2 was lower in the EV + H_2_O_2_ group (*P* < 0.05, Fig. 6a and c). However, TFEB overexpression attenuated the decrease in protein abundance of Bcl-2 induced by H_2_O_2_ (Fig. 6a and c). Furthermore, protein abundance of Bax, caspase 3 and caspase 9 was greater in the EV + H_2_O_2_ group compared with the EV group (*P* < 0.05, Fig. 6a, b, d and e). TFEB overexpression attenuated the increase in protein abundance of Bax, caspase 3 and caspase 9 induced by H_2_O_2_ (Fig. 6a, b, d and e). Consistent with alterations in the caspase apoptotic pathway, H_2_O_2_ treatment increased cellular apoptosis, while TFEB overexpression attenuated the increase in cellular apoptosis induced by H_2_O_2_ (*P* < 0.05, Fig. 6f).

## Discussion

Bovine mammary epithelial cells during early lactation are highly susceptible to altered redox (oxidation reduction) balance that can result in cellular oxidative damage. Autophagy is an evolutionarily conserved catabolic process that degrades cytoplasmic damaged organelles, long-lived proteins, or misfolded proteins to preserve cellular homeostasis and defend against oxidative or proteotoxic stress [21]. Upon induction of autophagy, a cytosolic form of LC3 (LC3-I) is conjugated to phosphatidylethanolamine to form LC3-phosphatidylethanolamine conjugate (LC3-II), which is recruited to autophagosomal membranes [22]. The protein p62 can bind to ubiquitinated protein aggregates and deliver them to autophagosomes, and itself is degraded by autophagy [23]. Thus, autophagic activity is positively correlated with the protein abundance of LC3, while inversely correlated with the protein abundance of p62 [24]. Previous studies in non-ruminants revealed that ROS stimulates cellular autophagy [25, 26]. In the present study, the greater intracellular ROS, greater protein abundance of LC3-II along with lower protein abundance of p62 underscored the positive effect of oxidative stress on autophagy in bovine mammary epithelial cells.

Autophagic flux occurs in sequential steps: biogenesis of phagophore membranes, selective or non-selective engulfment of cytoplasmic material into autophagosomes, and fusion with lysosomes to form autolysosomes [27]. Thus, upregulation of yellow puncta (autophagosomes) and red puncta (autolysosomes) in H_2_O_2_ cultures further confirmed activation of autophagic flux in bovine mammary epithelial cells under oxidative stress. Work by Li et al. [18], reported that enhanced autophagy increased the responsive ability of mammary epithelial cells to hyperketonemia-induced metabolic stress in dairy cows during early lactation. Thus, our data confirmed previous work underscoring that activation of autophagy is a feedback mechanism for reducing oxidative or metabolic stress [15]. Work in non-ruminants demonstrated that impaired autophagy contributed to oxidative damage as well as the pathogenesis of oxidative stress associated diseases [28, 29]. BafA1, a specific inhibitor of vacuolar-type H^+^-ATPase, blocks the downstream step of fusion between the autophagosomes and lysosomes, resulting in accumulation of LC3-II and p62 [30, 31]. The fact that BafA1 was effective in attenuating the autophagic activity resulting from H_2_O_2_ challenge through inhibition of fusion between the autophagosomes and lysosomes, and upregulation of LC3-II and p62 suggested that in the absence of autophagic activity ROS accumulates and results in oxidative stress and cellular apoptosis [32].

Lysosomal biogenesis and autophagy are closely regulated by the transcription factor TFEB [33]. Under basal conditions TFEB is phosphorylated and dispersed in the cytosol and on the lysosomal surface. Upon environmental stress, TFEB is dephosphorylated and translocate to the nucleus where it regulates transcription of genes belonging to the Coordinated Lysosomal Expression and Regulation network [34]. In turn, these genes coordinate the sequential steps of autophagy, from cargo recognition and autophagosome formation to vesicle fusion and substrate degradation [35]. Thus, promoting TFEB translocation into the nucleus to activate the autophagy-lysosomal pathway may be a potential treatment strategy for oxidative damage.

At least in non-ruminants, the enhanced autophagic activity potential of ROS stems from its ability to induce TFEB translocation into the nucleus [36, 37]. The fact that cultures with H_2_O_2_ increased nuclear protein abundance of TFEB and promoted TFEB movement into the nucleus is consistent with previous findings in SH-SY5Y cells in which H_2_O_2_ promoted TFEB nuclear translocation [38]. Furthermore, knockdown of TFEB by siRNA reversed the increase in protein abundance of LC3-II and the number of autophagosomes and autolysosomes induced by H_2_O_2_, indicating that TFEB mediate the elevated autophagic activity induced by oxidative stress. The specific mechanisms whereby activated TFEB travels to the nucleus and activates the transcriptional program in bovine mammary epithelial cells remain to be elucidated. From a regulatory standpoint it is noteworthy that mechanistic target of rapamycin (mTOR), an atypical Ser/Thr kinase in nonruminants, can directly regulate the expression of TFEB phosphorylation and nuclear translocation [39]. Martina et al., reported that inhibition of mTOR by rapamycin increases nuclear translocation of TFEB that activates autophagy [40]. Thus, we speculate that enhanced nuclear translocation of TFEB was partly due to inhibition of mTOR in bovine mammary epithelial cells. In fact, the phosphorylation of mTOR was lower in the mammary gland of dairy cows under metabolic stress [18].

Given the consequences of ROS accumulation on organelles and genomic integrity, autophagy is believed to be enhanced as a means to mitigate oxidative stress [41]. By promoting lysosomal secretion, TFEB overexpression in mouse embryonic fibroblasts increased number of autophagosomes and autophagic flux, generation of new lysosomes, and led to clearance of storage material in several lysosomal storage disorders [42]. Thus, the upregulation of LC3-II and downregulation of p62 protein abundance in response to overexpression of TFEB confirms that this transcription regulator plays a similar role in bovine mammary epithelial cells in promoting autophagic flux. In turn, enhanced autophagic flux ameliorates oxidative stress by downregulating ROS levels [43]. The decrease in intracellular ROS levels due to TFEB overexpression in the present study agrees with previous work in nonruminants [43] demonstrating a cytoprotective role of autophagy against oxidative stress.

Oxidative damage to secretory cells due to their high metabolic load was proposed as contributing to the lactational decline of secretory cell numbers and milk yield [8]. Clearly, activated caspase 9/3 dependent apoptotic pathway contributes to this process. Accumulation of ROS caused mitochondrial dysfunction, leading to activation of caspase 9 and caspase 3 mediated cascade reaction, thereby resulting in apoptosis [19]. Furthermore, alternations in the protein abundance of Bcl-2 family proteins (pro-apoptotic Bax and anti-apoptotic Bcl-2) are also involved in the regulation of oxidative stress-induced cellular apoptosis [44]. In the present study, the downregulation of protein abundance of Bax, caspase 9 and caspase 3, and upregulation of protein abundance of Bcl-2 in H_2_O_2_-challenged cells that were overexpressed with TFEB underscored the positive effect of autophagy on apoptotic pathways. Along with the downregulation of apoptotic pathways, the downregulation of cellular apoptosis in cultures with TFEB overexpression followed by H_2_O_2_ stimulation support the role of TFEB-mediated autophagy in dampening cellular apoptosis.

## Conclusions

The present findings indicate that H_2_O_2_ induces ROS accumulation in bovine mammary epithelial cells, which triggers TFEB nuclear translocation resulting in autophagy induction. By reducing ROS production and inhibiting caspase-dependent apoptosis, overexpression of TFEB promotes cellular autophagic flux that alleviates H_2_O_2_-induced oxidative damage. Taken together, the present study confirmed that TFEB-mediated autophagy may be a promising therapeutic target for reducing oxidative stress-triggered bovine mammary epithelial cells damage in the future.

## Data Availability

All data generated or analyzed during this study are included in this published article.

## Abbreviations

TFEB: Transcription factor EB
BafA1: Bafilomycin A1
ROS: Reactive oxygen species
siRNA: Small interfering RNA
DMEM/F-12: Dulbecco’s modified eagle’s medium nutrient mixture F-12 medium
DMSO: Dimethylsulfoxide
EV: Empty adenovirus vector
Ad-TFEB: TFEB overexpression adenovirus
DAPI: 4′,6-Diamidino-2-phenylindole dihydrochloride
PBS: Phosphate-buffered saline
PVDF: Polyvinylidene difluoride
TBST: Tris-buffered saline
ECL: Enhanced chemiluminescence solution
mTOR: Mechanistic target of rapamycin
